# IBN NAFIS – A FORGOTTEN GENIUS IN THE DISCOVERY OF PULMONARY BLOOD CIRCULATION

**Published:** 2010

**Authors:** M Akmal, M Zulkifle, AH Ansari

**Affiliations:** *National Institute of Unani Medicine, Bangalore, India*

**Keywords:** Pulmonary circulation, Unani Medicine, Ibn Nafis

## Abstract

Scientific theories take centuries to come into existence and they keep on evolving. Uncountable intellectual minds work on these theories; some fail to do anything about it; some add a little after tremendous efforts, and some people give remarkable and unforgettable contribution.

As far as credit is concerned, the person who is able to prove the theory by his facts and who clears the maximum doubts by his observations, experimentations, facts and reasoning, gets the credit for that theory, and this should be done with honesty.

The theory of pulmonary circulation took more than 2000 years to come into existence as we know it today. With the passage of time different people were given credit. Some say that it was given to Galen; some say it was Michael Servetus; others say that Realdus Columbus was the real discoverer; some gave the credit to Ibn Nafis, and finally people gave the credit to William Harvey. But after the rediscovery of Ibn Nafis’ manuscript no.62243 titled Sharah al Tashreeh al Qanoon, or “Commentary on the anatomy of Canon of Avicenna” in 1924 AD in Europe, it became clear that Ibn Nafis had described the pulmonary circulation almost 300 years before Harvey, and the historians like Aldo Mieli, Max Mayrhoff, Edward Coppola etc. clearly state that Ibn Nafis is the real discoverer of the pulmonary circulation and that he should be given the credit for the discovery of the pulmonary circulation.

## Introduction

When we eat a piece of bread, do we ever think for a moment how many people worked to bring it to us – from soaking a small seed to the formation of flour and then into a bread. It is just a matter of one or two years. But scientific theories take centuries to come into existence and they keep on changing; uncountable intellectual minds work on these theories. Some fail to do anything about it; some add a little after tremendous efforts, and some people give remarkable and unforgettable contribution.

As far as credit is concerned – “who was the first” – the best in the series of events gets the credit for the theory. But the person who gets the credit is not the only one who thought in that direction, but, he is the only one who is able to prove the theory by the facts and who clears the maximum doubts by his observations, facts and reasoning, although he is just a small part of that chain.

The same is true in the case of the discovery of the pulmonary circulation. There are so many names who contributed in this regard. But the person who clears the maximum doubts with the least resources should be given the real credit. As far as the discovery of pulmonary circulation is concerned, there is a series of people to whom credit was given by different people. Like, some say, it was Galen (129-200AD) who discovered the pulmonary circulation; some say it was Ibn Nafis (1210-1288AD) who conceived the idea; still some thought that Michael Servetus (1511-1553AD) was the real one and some thought about Realdus Columbus (1516-1559AD) and finally, the people of the modern era gave the real credit to William Harvey (1578-1657AD).

Here we will attempt to identify to whom actual credit for discovering the pulmonary circulation should be given to by comparing their postulates and contributions to the theory of the pulmonary circulation.

## History of pulmonary circulation at a glance

The table below summarizes the physicians throughout history who contributed to our understanding of the pulmonary circulation. It really starts with Hippocrates and ends with Marcello Malpighi…

**Table:** 

Hippocrates (Buqrat)	460-377 BC
Aristotle (Arastu)	384-322 BC
Erasistratus (Aerasistratoos)	290 BC
Galen (Jalinus)	129-200 BC
Annafis (Ibne Nafis)	1210-1288 AD
M. Servetus	1511-1553 AD
Andreas Vesalius	1514-1564 AD
Realdus Columbus	1516-1559 AD
Caesalpinus	1519-1603 AD
Fabricius de Aquapendente	1537-1619 AD
William Harvey	1578-1657 AD
Marcelo Malpighi	1628-1698 AD

## Postulates of various ancient physicians about the circulation:

### Hippocrates (*Buqrat*; 460-377 BC):

Hippocrates, the Father of Medicine, believed that the liver and the spleen were the central organs within which blood travelled to the heart to be warmed or cooled by the air entering the lungs and the heart via the trachea.

### Aristotle (*Arastu*, 384-322 BC):

Attributed three ventricular chambers to the heart; he named the main artery of the heart as Aorta.

### Erasistratus (330/304/290 BC):

Arteries contain air. He said air from the lungs goes to the heart and changes into vital spirit. He gave the names of vessels, like artery, vein, pulmonary artery and pulmonary vein, etc.

Concept of reverse circulation: *“The blood which oozes out through artery when cut comes from veins through very small vessels between artery and vein”.*

### Galen (Jalinus, 129-200 AD):

In his book *“De Usu Partium”* Galen wrote: *Arteries carried blood instead of air.* Veins as well as arteries both carry blood towards the extremities. According to Galen, there were two kinds of blood: Spiritual blood (arterial blood, present in left heart); venous blood (present in right heart). Spiritual blood nourishes light and delicate texture organs e.g. lungs, and venous blood nourishes those of heavy and gross texture organs e.g. liver.

About arteries he said that they have the property to pulsate i.e. *pulsative faculty or pulsific virtue.* There are certain openings in the interventricular septum through which some amount of blood goes from right side to left side of the heart. Galen placed the seat of sanguification in the liver. [Sanguification = conversion of blood = hematopoiesis - Ed.]

Galen believed that blood was propelled inside the vessels by attraction from peripheral tissues in need of nutrition (he did not recognized the pumping action of the heart). Galen believed that blood perfuse into the organs like water in irrigating fields.

Galen could not explain how blood produced in the liver and carried to the right heart by vena cava reached the left heart so that it could be distributed to the arterial tree.

Galen had a good notion of the aortic, pulmonary and cardiac valves and said that arteries and veins communicate by common openings which are invisible and extremely narrow.

### Ibn Nafis (1210-1288 AD):

Ala ad-Din Abu al-Hasan Ali Ibn Abi-Hazm-al-Qarshi known as *Ibn Nafis Damishqi*, was born in a small town near Damascus called Qarsh. He is considered as the ***Father of Circulatory Physiology.*** In 1236 AD he moved to Egypt, worked in *Almansouri Hospital* and became the chief of physicians and the Sultan’s personal physician there. He wrote many books in medicine but his most famous book was *Sharah al Tashreeh al Qanoon **(Commentary on anatomy of the Canon of Avicenna)**.* This book was forgotten until 1924 when an Egyptian physician, Dr M. Altatawi discovered manuscript No.62243 titled “Commentary on the anatomy of the Canon of Avicenna” in the Prussian state Library in Berlin, Germany. This book contains the first description of the pulmonary circulation.

### The Commentary stated:

- Blood from the right chamber of the heart to the left chamber does not come through direct pathway.
- The interventicular septum does not have visible or invisible pores.
- “The lungs are composed of parts, one of which is the bronchi, the second the branches of the arteria venosa and the third the branches of the vena arteriosa, all of them connected by loose porous flesh”.
- Blood from the right chamber of the heart goes to -vena arteriosa (pulmonary artery) - lungs - arteria venosa (pulmonary vein) - left chamber (here the vital spirit is formed).
- “His [Avicenna’s] statement that the blood that is in the right side nourishes the heart is not true at all, for nourishment of the heart is actually from the blood that goes through the vessels that permeate the body of the heart.” This particular passage put forward the concept of coronary circulation.

### Michael Servetus (1511-1553 AD):

Michael Servatus or in Spanish, Miguel Serveto, a Spanish anatomist and theologian, in his theological book “*Christianismi Resstitutio*” in 1553 wrote: “air mixed with blood that is sent from the lungs to the heart through the arterial vein; therefore, the mixture is made in the lungs. The bright color is given to the sanguine spirit by the lungs, not by the heart.

“*The communication does not take place through the median partition of the ventricle, as is generally supposed, but by a long and wonderful route; the blood is conducted through the lungs where it is agitated and prepared and where it becomes yellow and passes from arterial vein into the venous artery”*. Blood goes to lungs not only for nourishing it but also for air mixing.

Since the book was a theological work and condemned by most of the Christian factions of his time, he was burnt alive at the stake, in Geneva in 1553 AD, on order given by John Calvin, the French Protestant theologian. It also seems doubtful that this concept of the pulmonary circulation was from his own observations. He might have come across the writings of Ibn Nafis while he was assistant professor to Prof. John Guinter who translated most of Galen’s work and those of other physicians from antiquity as well as several Arabic manuscripts of the Middle Ages into Latin.

### Postulates of Realdus Colombus (1516-1559 AD):

Six years after Servetus, one of the best Anatomists in Padua University, an Italian physician, Realdus Colombus, discovered again and independently the pulmonary circulation. He said in his book *De Re Anatomica* in 1559 AD “*between these ventricles there is a septum through which almost everyone believes that there opens a pathway for the blood from the right ventricle to the left ventricle and the blood is rendered thin so that this may be done more easily for the generation of vital spirits, but, they are in great error,* ......”

His theory of pulmonary circulation was probably based on Servetus writings but does not cite Servetus.

### Postulates of Caesalpinus (1519-1603):

An Italian physician, philosopher, and most distinguished botanist of his time, he studied at University of Pisa. He also described the pulmonary circulation without knowing Realdus Colombus. Caesalpinus formally named the passage of the blood from the right side of the heart to the left side via the lungs, *the circulation*.

Caesalpinus was the first and only one who has drawn attention towards the swelling of the vein which takes place below and never above a ligature.

### Fabricius Ab Aquapendente (1537-1619):

He was an Italian surgeon and anatomist and studied at the University of Padua. *He was the teacher of Harvey. He discovered valves of veins which he described in his book De venarum ostiolis*.

### Andreas Vesalius (1514-1563):

He is said to be the father of modern anatomy. He also described in his book *De Fabrica humani corporis*, the pulmonary circulation in a manner similar to Ibn Nafis. More importantly and interestingly in the first edition of his book, Vesalius agreed with Galen that blood “soaks plentifully through the septum from the right ventricle into the left”. But in the second edition, he omitted the above statement and instead wrote “I still do not see how even the smallest quantity of blood can be transfused through the substance of the septum from the right ventricle to the left ventricle.”

### Postulates of William Harvey (1578-1657):

It is said that Harvey in 1616 AD expounded on those original and complete views of the circulation of the blood and in 1628 AD he gave his views to the world in his celebrated treatise “*Exercitatio Anatomica de Motu Cordis et Sanguinis in Animalibus*” (an anatomical study of the motion of the heart and of the blood in animals.

In his treatise [Harvey’s] he proved:

- That it is the contraction, not the dilatation, of the heart which coincides with the pulse, and that the ventricles as true muscular sacs squeeze the blood which they contain into the Aorta and Pulmonary artery.
- That the pulse is not produced by the arteries enlarging and so filling, but by the arteries being filled with blood and so enlarging.
- That there are no pores in the septum.
- That the blood in the arteries and in the veins is the same blood.
- That the action of the right and left sides of the heart, auricles, ventricles and valves, is the same, the mechanism in both being for reception and propulsion of liquid and not for air, since the blood on the right side, thoroughly mixed with air is still blood.
- That the blood sent through the arteries to the tissues is not all used, but that most of it runs through into the veins.
- That there is no to and fro undulation in the vein but a constant stream from the distant parts towards the heart.
- That the dynamic starting point of the blood is the heart and not the liver.

Harvey was not able to recognize the capillary channels by which the blood passes from the arteries to the veins. However he did not understand the physiology of the pulmonary circulation-dissipation of the CO_2_ and replacement with O_2_ which was fully elucidated by Lavoisier in the 18th century.

### Marcello Malpighi (1628-1698AD):

He discovered capillaries in 1660-61 and thereby discovering the capillary circulation. So, therefore, he solved the millennia-old mystery of how blood goes from the arteries to the veins.

## Conclusion

At this point we can try to answer the question: ***Who was the real discoverer of the pulmonary circulation***? There are four possible contenders:

- **Galen** - The passage, through the lungs of some part (*no matter how little*) *of the blood of the right ventricle into the left ventricle.*
- **Ibn Nafis** - *The passage through the lungs, of a large part (or most) of the blood of the right ventricle into the left ventricle.*
- **Servetus** - *The passage through the lungs (where it is mixed with air), of all blood of the right ventricle into the left ventricle*.
- **Colombus** - *The reception of blood from the vena cava through the lungs where it is “prepared” and its reception by the left ventricle from which, when the heart is constricted, it is distributed to the whole body.*

## Views of a few Modern Historians

### 

#### Aldo Mieli

“We believe that henceforth it is fair to attribute the discovery of the pulmonary circulation to **Ibn Nafis** who was a distant precursor of the physicians of the sixteenth century Italian school and of William Harvey who, four centuries later, described the whole of the pulmonary circulation in an accurate, clear and definitive manner.

#### Max Meyrhoff

A distinguished scholar of the history of Arabic Medicine stated: “We have seen that **Ibn Nafis**, three centuries before Colombus, had already noticed visible passages between the two types of pulmonary vessels.”

#### Edward Coppola

In the William Osler Medical Essay on the discovery of the pulmonary circulation, Edward Coppola said: “The theory of pulmonary circulation propounded by **Ibn Nafis** in the 13th century was not forgotten and that centuries after his death it may have influenced the direction of the anatomical investigations of Colombo and Valverde, who finally announced it to the Western world as a physiological fact susceptible to experimental proof.

**Sami Haddad** from Lebanon published an article in the annuals of surgery in 1936 AD about Ibn Nafis and other articles were also published by **Ayman et al** and **Dr Abdul Karim Shahadah** from Syria which showed clearly that **Ibn Nafis** should be given the credit for the discovery of the pulmonary circulation 300 years before even William Harvey was born.

**Dr Abdul Rehman** in his article, titled “the discovery of the blood circulation” also proved it: “*In 1242 Ibn Nafis was the first to describe human blood circulation and pulmonary circulation.*”

Thus, in agreement with **Sarton**, it is evident from this study that Ibn **Nafis** is the greatest physiologist of the Middle Ages and the main forerunner of Servetus, Vesalius, Columbus and Harvey in the description of the pulmonary circulation as we know it today. He was also a talented physician and a gifted medical writer. His discoveries and medical works greatly contributed to the progress of medical knowledge and the advancement of medical practice. His influence on the generation of doctors and scholars, who came after him both in the East and West, is well documented up to the 17^th^ Century.

So it is clear enough to understand, by the statements of so many intellectuals and modern historians that without any confusion **Ibn Nafis** should be given the credit for the discovery of the pulmonary circulation.
